# Accuracy of Coverage Survey Recall following an Integrated Mass Drug Administration for Lymphatic Filariasis, Schistosomiasis, and Soil-Transmitted Helminthiasis

**DOI:** 10.1371/journal.pntd.0004358

**Published:** 2016-01-14

**Authors:** Philip J. Budge, Edmond Sognikin, Amanda Akosa, Els M. Mathieu, Michael Deming

**Affiliations:** 1 Epidemic Intelligence Service, Centers for Disease Control and Prevention, Atlanta, Georgia, United States of America; 2 Parasitic Diseases Branch, Centers for Disease Control and Prevention, Atlanta, Georgia, United States of America; 3 Division of Community Health, Ministry of Health, Lomé, Togo; 4 Department of Biology, Georgia State University, Atlanta, Georgia, United States of America; The George Washington University School of Medicine and Health Sciences, UNITED STATES

## Abstract

**Background:**

Achieving target coverage levels for mass drug administration (MDA) is essential to elimination and control efforts for several neglected tropical diseases (NTD). To ensure program goals are met, coverage reported by drug distributors may be validated through household coverage surveys that rely on respondent recall. This is the first study to assess accuracy in such surveys.

**Methodology/Principal Findings:**

Recall accuracy was tested in a series of coverage surveys conducted at 1, 6, and 12 months after an integrated MDA in Togo during which three drugs (albendazole, ivermectin, and praziquantel) were distributed. Drug distribution was observed during the MDA to ensure accurate recording of persons treated during the MDA. Information was obtained for 506, 1131, and 947 persons surveyed at 1, 6, and 12 months, respectively. Coverage (defined as the percentage of persons taking at least one of the MDA medications) within these groups was respectively 88.3%, 87.4%, and 80.0%, according to the treatment registers; it was 87.9%, 91.4% and 89.4%, according to survey responses. Concordance between respondents and registers on swallowing at least one pill was >95% at 1 month and >86% at 12 months; the lower concordance at 12 months was more likely due to difficulty matching survey respondents with the year-old treatment register rather than inaccurate responses. Respondents generally distinguished between pills similar in appearance; concordance for recall of which pills were taken was over 80% in each survey.

**Significance:**

In this population, coverage surveys provided remarkably consistent coverage estimates for up to one year following an integrated MDA. It is not clear if similar consistency will be seen in other settings, however, these data suggest that in some settings coverage surveys might be conducted as much as one year following an MDA without compromising results. This might enable integration of post-MDA coverage measurement into large, multipurpose, periodic surveys, thereby conserving resources.

## Author Summary

Mass drug administration (MDA) is an important tool in elimination efforts for several neglected tropical diseases, including lymphatic filariasis (LF), onchocerciasis, trachoma, schistosomiasis, and soil-transmitted helminthiases (STH). The success of control and elimination programs depends upon achievement of target coverage levels during MDA. Community-based surveys can be used to verify coverage after an MDA, but recall accuracy in post-MDA coverage surveys has rarely been formally tested. To test recall accuracy, we compared survey responses among members of a population that received an integrated MDA for LF, onchocerciasis, schistosomiasis, and STH in a series of coverage surveys to verified MDA treatment records. Coverage estimates based on survey responses were highly consistent between samples surveyed at 1, 6, and 12-months (range 88–91%) and concordance for any ingestion of MDA drugs was >86% in all surveys. Furthermore, respondents were able to identify which of the three MDA medications they took with up to 80% accuracy. These findings suggest that in some settings coverage surveys can provide consistent information up to a year following an integrated MDA and should be considered as a tool for primary assessment of coverage as well as for validating reported coverage.

## Introduction

Preventive chemotherapy (PCT) is used at a population level for seven neglected tropical diseases (NTDs): lymphatic filariasis (LF), onchocerciasis, trachoma, schistosomiasis, and the soil-transmitted helminthiases (STH) [1,2]. PCT is provided via mass drug administration (MDA), usually conducted annually, during which drug distributors disperse medications en masse to the target populations at risk for each disease, except where contraindicated due to young age, pregnancy, or illness. Coverage, the percentage of the defined target population that takes the pills offered at MDA, is an essential indicator of the success of treatment programs and WHO guidelines mandate reporting of coverage as part of monitoring and evaluation of program activities.[2] WHO guidelines state that coverage should be based on directly observed swallowing of the tablets delivered and reported or “administrative” coverage in mass drug distributions is generally calculated as the number of persons treated (as reported by drug distributors), divided by the estimated size of the target population. Accuracy of reported coverage is therefore dependent on the completeness and accuracy of the distributors’ reports and on the estimate of population size; inaccuracies in reported coverage can occur when either or both of these estimates are erroneous.

Alternatively, coverage can be measured using cluster-sample surveys similar in purpose to those used by the Expanded Programme on Immunization (EPI) [3]. Community-based cluster surveys have several advantages as measurements of MDA coverage [4,5]. (1) They are independent of drug distributors, who may have financial or other incentives to over-report coverage. (2) They do not rely on estimates of population size, such as potentially outdated or inaccurate census data [6]. The can also (3) collect information on knowledge, attitudes, and practices, and (4) help monitor adherence of drug distributors to programmatic guidelines (for example, whether distributors directly observed the pills being swallowed).

Despite these advantages, coverage surveys are infrequently used for validation of MDA coverage, in part because of the resources (human and financial) needed to conduct them, but also because of concerns about susceptibility to recall and reporting bias. There are few published data regarding accuracy of respondent recall. Estimates of mothers’ recall accuracy for infant vaccination have yielded disparate results [7–9]. It is unclear whether, and under which circumstances, self-report of receipt of treatment (vaccination or drug administration) is acceptably accurate for use as a measurement of drug (or vaccine) administration.

To complicate matters, receipt of PCT is becoming more complex as MDAs for multiple NTDs are integrated to maximize efficiency of distribution. Because target coverage levels vary amongst the NTDs targeted by an integrated MDA [2], it may be necessary to estimate drug-specific coverage to satisfy reporting requirements. To do this using post-MDA coverage surveys, one might need to ask about not only whether MDA medications were taken, but also which medications (in the event a respondent reported taking some, but not all). The accuracy of such results would depend on the ability of respondents to accurately recall whether they took MDA medications and which ones they took.

To test the accuracy of respondent recall following an integrated MDA, we conducted community cluster-sample surveys following the first triple-drug integrated MDA in Togo in 2008.

## Methods

### Study design

The overall aim of the study was to test the recall of participants in the MDA by carefully recording each person receiving MDA medications, then visiting a sample of extended-family dwellings (compounds) at 1, 6, and 12 months following the MDA to conduct coverage surveys. After the surveys, data from the MDA registers and the survey responses were compared to determine recall accuracy. A secondary aim was to measure the adherence of drug distributors to MDA guidelines.

### Ethics statement

This project was submitted for human subjects review to the Center for Global Health at the Centers for Disease Control and Prevention (CDC), Atlanta, Georgia, USA. The project was determined to be program evaluation under CDC policy prior to the implementation of the survey. Permission for the survey was obtained from the Togolese Ministry of Health.

### Study area

Kémérida Canton, one of 11 cantons within Binah District, Togo, is located along the northeastern border of Togo. Based on the population of enumeration areas that later constituted Kémérida Canton in census spreadsheets dated 2004, the population of the canton was 4,488. This site was selected as the study area due to its manageable size, making it possible to enumerate all compounds. Compounds consisted of one or more households, often belonging to members of an extended family, and usually surrounded by a wall or some other barrier. All compounds in Kémérida Canton were enumerated and marked with a number, usually on the compound wall or door. Information collected for each compound included the chief of compound’s name and number of households in the compound. All households belonged to one and only one compound.

### Sampling strategy

All compounds in Kémérida Canton were eligible for selection to participate in the recall surveys. A cluster sampling survey design was used, with compounds as the primary sampling units. Prior to the first survey, compounds were systematically selected for inclusion in each of the three planned surveys from a line-listing of all compounds in the Canton using a fixed sampling interval and a random starting point. For the 1-month survey, every eighth compound was selected after selecting a random start among the first eight compounds. For the 6 and 12-month surveys, every fourth compound was selected, each starting with a randomly selected compound among the first four compounds that would not lead to the inclusion of any compounds selected for a prior survey round. Study staff interviewed all members of all households within each selected compound, with mothers or another adult answering providing information for children <10 years of age.

### MDA distribution

MDA medications were distributed house-to-house throughout Binah District in May 2008 by community health workers (CHWs) employed by the Ministry of Health. Ivermectin, albendazole, and praziquantel were distributed according to age- and height-based WHO dosing guidelines [2] (Table 1). CHWs were instructed to directly observe drugs being swallowed and to record the name, age, and gender of all treated individuals in a treatment register, according to national MDA protocol. To ensure the accuracy of the treatment registers, a trained member of the study team accompanied CHWs within the study area (Kémérida Canton). These observers were literate members of the local community, often school teachers. The observers’ primary role was to ensure that the medication doses for all persons treated were accurately recorded in the MDA register. To minimize the effect their presence might have on recall, they did not actively participate in either drug distribution or education.

**Table 1:** Drug doses and inclusion criteria during integrated MDA in Binah District, Togo, 2009. Exclusion criteria for all drugs included pregnancy or severe illness.

MDA Drug	Inclusion Criteria	Dose Range (# of Tablets)	Diseases Targeted
Albendazole	≥ 2 years of age	½ to 1	Lymphatic filariasis, Soil-transmitted helminthiasis
Ivermectin	≥ 90 cm	1 to 4	Lymphatic filariasis, Onchocerciasis
Praziquantel	≥ 94 cm	½ to 5, by halves	Schistosomiasis

### Coverage surveys

**Fig 1:**
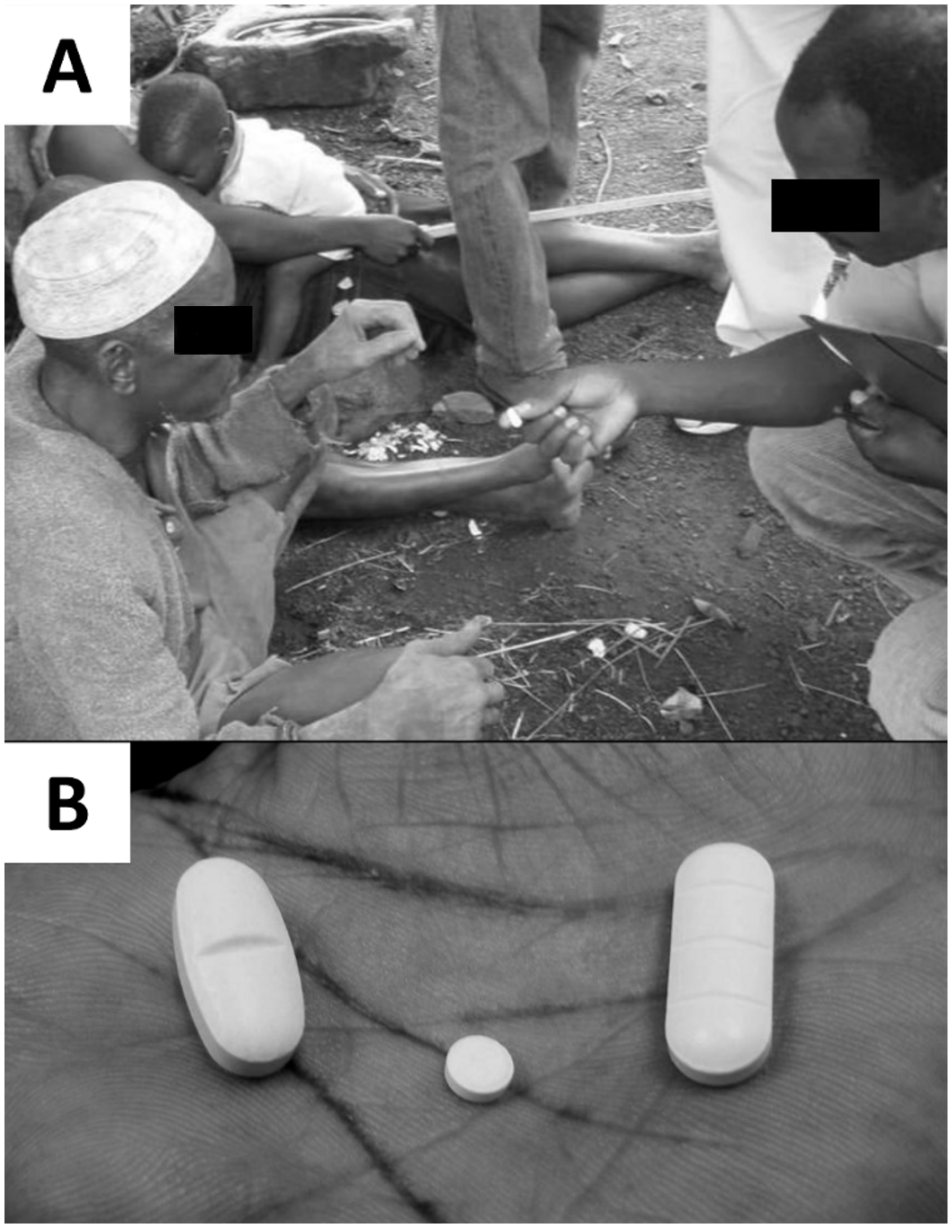
The recall interview. A) A typical interview setting. Interviewers visited each compound, showed examples of the pills that had been given during the MDA, and asked respondents if they had taken each pill. B) Pills given during the MDA, from left to right: albendazole, ivermectin, praziquantel.

Surveys were conducted at 1, 6, and 12 months following the MDA. Interviewers were residents of the local community, who were literate in French and Kabiyé, the local language. All interviewers took part in a 5-day training prior to the first survey, which provided an overview of the study design, sampling technique, and the survey instrument and included simulated interviews with trainers and practice interviews with volunteer household members. Interviewers also attended a single day of refresher training prior to each subsequent survey. Interview forms were written in French and interviews were conducted in either French or Kabiyé. Accurate translation of study questions was verified by back-translation of Kabiyé versions. Interviewers asked respondents whether they had been offered pills during the May, 2008 MDA and whether they swallowed the pills they were offered. If the respondent reported swallowing MDA medications, the interviewer showed an example of each pill and allowed the respondent to hold it, and then asked if that pill had been taken, and if yes, how many (Fig 1).

### Survey instrument and treatment register

he survey instrument was a 2-page, tabular form for each household that allowed responses for up to 12 household members. To facilitate compilation of treatment and recall data, the form was organized as a roster of household members, with separate column sections to record survey responses and, subsequently, treatment information from the MDA register. Slight modifications were made to the survey instrument after each survey, to improve clarity. After interviewing all members of selected households, interviewers located the treatment register from the May 2008 MDA, and located the information listed for the respondents they had interviewed. The actual treatments were then transcribed from the MDA register onto the appropriate section of the interview form. This allowed compilation of both recall and treatment information for each household on a single form, while leaving the treatment register in the hands of the community health workers or in storage at health facilities.

### Data entry and analysis

After each survey, data were double-entered using CSPro (US Census Bureau, Washington DC)[10]. Discrepancies were resolved by review of the primary data forms. Data cleaning and analysis were performed using SAS version 9.2 (Cary, NC) and STATA version 12.1 (College Station, TX). The analysis was weighted according to the differential probabilities of selection, and the standard errors took into account the cluster-sample design and the high sampling fractions. For concordance calculations, concordant responses were defined as a “yes” or “no” survey response that agreed with documentation of treatment in the MDA register. Missing or “don’t know” responses were included in the denominator as non-concordant. When calculating concordance for recall of pill numbers, ½ tablets were rounded up.

### Multivariable logistic regression analysis

To explore potential risks for inaccurate survey responses to the question of overall MDA participation (“did you take at least one of the MDA medications”), we conducted a multivariable logistic regression analysis that pooled responses from all surveys, accounting for clustering by compound, the primary sampling unit (SAS procedure “surveylogistic”, SAS version 9.2, Cary, NC). All relevant available variables were included in the model: age (at the time of the survey), gender, self-reported pregnancy status at the time of MDA, and survey date (i.e. 1, 6, or 12 months).

## Results

### Study population

Prior to the study, all compounds in Kémérida Canton were enumerated and line listed. From among the 413 compounds in the Canton, 51 were systematically selected for inclusion in the 1-month survey, 104 for the survey at 6 months, and 103 for the survey at 12 months. A total of 598, 1,335, and 1,073 persons were interviewed for the 1-, 6-, and 12-month surveys, respectively (Table 2). Survey data could not be collected for 12% (1 month), 14% (6 months), and 3% (12 months) of residents of the responding compounds due to the unavailability of individual residents (or, for those aged <10 years, a responsible adult) at the time the survey was conducted. Another 3% (1 month), 1% (6 months), or 9% (12 months) of those surveyed were excluded because they reported not living in their current household at the time the MDA was distributed. In total, responses were analyzed for >85% of the residents of participating compounds (Table 2). Demographics for those living in Kémérida at the time of the 2008 MDA but not available for interview during the coverage surveys can be found in the supplemental material (S1 Table).

**Table 2:** Characteristics of each survey sample.

	Survey			
	1 Month	6 Months	12 Months	P value*
Compounds selected	51	104	103	
Compounds interviewed	45 (88%)	99 (95%)	94 (91%)	
Total households in interviewed compounds	107	244	190	
Total residents in interviewed compounds	598	1335	1073	
Residents unavailable for interview	73 (12%)	188 (14%)	32 (3%)	**<0.001**
Residents not present at time of MDA	19 (3%)	16 (1%)	94 (9%)	**<0.001**
Total survey responses	506 (85%)	1131 (85%)	947 (88%)	**0.026**
Percent male	44	48	49	0.183
Median age in years (IQR)	17 (7–36)	19 (8–35)	21 (9–37)	**0.017**
Number aged <10 years	185 (37%)	356 (31%)	257 (27%)	**0.001**

*Chi^2^ for tests of proportion, Kruskall-Wallis for age.

### Coverage estimates

The survey tested several potential recall coverage indicators. Responses to the first three questions, which dealt with overall participation in the MDA (Table 3, “Overall coverage”) were highly consistent across surveys; >88% of persons in each survey indicated they were offered medication during the 2008 MDA and essentially the same proportion reported swallowing all the MDA medications they were offered. For the 1-month survey, the proportion reporting taking at least one MDA medication was almost exactly the same as the proportion recorded as taking at least one medication in the MDA register. While overall recall responses were consistent across surveys, the percentage of respondents with treatment documented in the MDA register declined (Table 3, “Treatment documented in MDA register”). The survey teams reported difficulty finding records for many surveyed individuals and households at the 6 and 12 month surveys. Since persons not appearing in the MDA register were presumed to be untreated during the MDA, the increasing difficulty of locating all treatment records in the 6 and 12 month surveys likely caused the documented coverage in these surveys to be spuriously low. There was less consistency between surveys when respondents were asked to identify which pills they swallowed (Table 3, “Medication specific coverage”).

**Table 3:** Drug coverage from the May, 2008 mass drug administration (MDA) according to survey responses and the MDA register.

	Percent coverage estimate (95% confidence interval)*		
Coverage Indicator	1 Month (N = 506)	6 Months (N = 1131)	12 Months (N = 947)
Overall coverage			
"Did a community health worker carrying a stick to measure height offer you white pills in May, 2008?"	88.5 (85.6–91.5)	91.8 (90.2–93.3)	90.0 (88.3–91.7)
"Did you swallow at least one of the pills the community health worker offered you?"	87.9 (84.8–91.1)	91.4 (89.8–93.0)	89.4 (87.6–91.2)
Did you swallow all the pills the community health worker offered you?"	87.5 (84.3–90.7)	91.1 (89.5–92.6)	89.3 (87.5–91.1)
Medication-specific coverage			
"Did you take this oval pill with one line?" (albendazole)	73.7 (64.6–82.9)	89.4 (87.7–91.0)	84.8 (82.7–86.9)
"Did you take this small round pill?" (ivermectin)	76.1 (67.7–84.5)	86.4 (84.6–88.2)	85.9 (83.8–87.9)
"Did you take this long rectangular pill with 3 lines?" (praziquantel)	71.7 (62.7–80.8)	85.6 (83.8–87.4)	82.3 (79.7–84.8)
Treatment documented in MDA register			
Received at least one medication	88.3 (85.8–90.8)	87.4 (84.7–90.0)	80.0 (76.3–83.7)
Received albendazole	88.3 (85.8–90.8)	87.3 (84.6–89.9)	78.4 (74.5–82.2)
Received ivermectin	85.8 (83.1–88.5)	83.5 (81.3–85.8)	76.6 (73.1–80.0)
Received praziquantel	85.4 (82.4–88.4)	82.2 (80.1–84.4)	74.3 (70.8–77.8)

* Coverage was defined as the number of persons in the population who took the medication, therefore all results represent the number who took or reported taking each medication, divided by the total population. Those who responded "don't know" or who refused to answer a particular question are included in the denominator.

### Concordance analysis

Comparing survey responses to treatment documented in the MDA register, concordance for taking at least one MDA medication was 95.3% for the 1 month survey, 92.4% for the 6 month survey, and 86.1% for the 12 month survey (Table 4). At one month, the discordant answers were equally distributed among those who erroneously reported taking at least one medication (2.2%) and those who erroneously reported taking no medication (2.2%). The proportion of respondents reporting taking MDA medications but for whom no documentation was found in the MDA register was 2.5%, 5.0%, and 11.5% in the 1, 6, and 12 month surveys, respectively. Concordance for individual pill recall was generally lower than for overall coverage. Concordance for taking ivermectin was slightly higher (86.0 and 84.9 percent, respectively) than for albendazole (81.8 and 83.3 percent) or praziquantel (81.6 and 83.3%) in the 1- and 12-month surveys, but these differences did not reach statistical significance. Recall accuracy for taking each individual medication was best at the 6-month survey (Table 4).

**Table 4:** Detailed comparison of survey responses compared to treatments recorded in MDA register.

	Treatment documented in MDA treatment register								
	1 Month			6 Months			12 Months		
Question	No	Yes	Unclear	No	Yes	Unclear	No	Yes	Unclear
“Did you swallow at least one of the pills the CHW offered you?”									
Survey responses									
No	48 (9.5%)	11 (2.2%)	0	73 (6.5%)	14 (1.2%)	4 (0.4%)	77 (8.1%)	16 (1.7%)	0
Yes	11 (2.2%)	434 (85.8%)	0	57 (5.0%)	972 (85.9%)	5 (0.4%)	109 (11.5%)	738 (77.9%)	0
Uncertain*	0	2 (0.4%)	0	4 (0.4%)	2 (0.2%)	0	3 (0.3%)	4 (0.4%)	0
Percent concordant (95% CI)	95.3 (93.3–97.2)			92.4 (89.1–95.7)			86.1 (82.3–89.9)		
“Did you take this oval pill with one line (albendazole)?"									
Survey responses									
No	48 (9.5%)	17 (3.4%)	0	83 (7.3%)	14 (1.2%)	4 (0.4%)	88 (9.3%)	24 (2.5%)	0
Yes	7 (1.4%)	366 (72.3%)	0	38 (3.4%)	968 (85.6%)	5 (0.4%)	102 (10.8%)	701 (74.0%)	0
Uncertain*	4 (0.8%)	64 (12.6%)	0	14 (1.2%)	5 (0.4%)	0	15 (1.6%)	17 (1.8%)	0
Percent concordant (95% CI)	81.8 (74.7–89.0)			92.9 (90.1–95.7)			83.3 (80.1–86.4)		
“Did you take this small round pill (ivermectin)?”									
Survey responses									
No	59 (11.7%)	10 (2.0%)	0	104 (9.2%)	10 (0.9%)	2 (0.2%)	100 (10.6%)	10 (1.1%)	0
Yes	9 (1.8%)	376 (74.3%)	0	47 (4.2%)	930 (82.2%)	0	109 (11.5%)	704 (74.3%)	0
Uncertain*	4 (0.8%)	48 (9.5%)	0	32 (2.8%)	5 (0.4%)	1 (0.1%)	13 (1.4%)	11 (1.2%)	0
Percent concordant (95% CI)	86.0 (79.5–92.4)			91.4 (88.8–94.0)			84.9 (81.5–88.3)		
“Did you take this long rectangular pill with 3 lines (praziquantel)?”									
Survey responses									
No	58 (11.5%)	21 (4.2%)	0	116 (10.3%)	9 (0.8%)	0	118 (12.5%)	16 (1.7%)	0
Yes	8 (1.6%)	355 (70.2%)	0	51 (4.5%)	917 (81.1%)	0	108 (11.4%)	671 (70.9%)	0
Uncertain*	8 (1.6%)	56 (11.1%)	0	34 (3.0%)	4 (0.4%)	0	17 (1.8%)	17 (1.8%)	0
Percent concordant (95% CI)	81.6 (74.6–88.7)			91.3 (88.9–93.7)			83.3 (80.0–86.6)		

*Uncertain survey responses included those marked “don’t know”, those where no response was given, and those where there was evidence of a changed response on the initial data form

To test the limits of respondent recall, we asked those who reported taking MDA medications to report how many of each tablet they took. Concordance for accurate recall of individual pill numbers was ranged from 36% (praziquantel, 12-month survey) to 85% (albendazole, 6-month survey, S2 Table).

### Compliance with MDA guidelines

To check compliance with MDA guidelines, we asked respondents who reported taking medications whether they swallowed them in the presence of the CHW. The percentages reporting swallowing the MDA medications in the presence of the CHW were 95.5%, 97.6%, and 96.5%, respectively, suggesting a high level of compliance with the MDA requirement for directly observed therapy (S3 Table). To check whether MDA treatment had been adequately reported, surveyors asked whether the CHW had been accompanied by a second observer (member of the study team) during the 2008 MDA; 100%, 88%, and 99% of those surveyed in the 1-, 6-, and 12-month surveys, respectively, reported the CHW was accompanied by an observer. We further checked CHW adherence to guidelines by evaluating coverage among children less than two years of age and pregnant women, groups that should have been excluded from receiving MDA medications. During the coverage surveys, between 7 and 11% of women aged 15–45 reported being pregnant at the time of the 2008 MDA (it is unclear how many of these reported the pregnancy to the CHW during the MDA); among these, 0–13% reported taking at least one MDA medication, and treatment was documented in the MDA register for 9–28%. Survey and MDA register results also indicate that between 2–12% of children <2 years during the MDA received albendazole (S3 Table).

### Data quality

Because survey and MDA register data were recorded on the same form, there was the potential for interviewers to inappropriately “correct” the survey responses to align them with the MDA register data; this possibility was suggested by the very high concordance for individual pill recall from the 6-month survey. Two of the authors reviewed each of the original survey forms and found that 11% of the 6-month survey responses had evidence of having at least one response erased and re-entered, compared to only 2% at 12 months and none at 1 month. Whether this indicates that the surveyors at 6 months intentionally “corrected” survey responses to bring them in to alignment with the MDA register is unclear. There were three surveyors who participated in the 6-month survey, with “correction” rates of 9.5%, 9.9%, and 16.4%, rates much higher than seen on the forms from 1 and 12 months. None of the three 6-month surveyors participated significantly in the 1-month or 12-month surveys.

### Predictors of concordance

To identify predictors of accurate recall, we examined the combined data from all three surveys by multivariable logistic regression analysis, controlling for survey, gender, and pregnancy status. Maternal recall for children <10 years of age was significantly less accurate than self-recall in other age groups (Table 5). Pregnancy status and the survey time point were also significantly associated with lower odds of concordance.

**Table 5:** Multivariable logistic regression analysis of the effect of age, gender, and self-reported pregnancy status on concordance. Odds ratio for concordance (95% CI).

	Did you take at least one pill?	Did you take this oval pill with one line?	Did you take this small round pill?	Did you take this long rectangular pill with 3 lines?
Survey				
1 Month	Ref	Ref	Ref	Ref
6 Months	**0.57 (0.38–0.86)**	**2.83 (1.82–4.43)**	**1.66 (1.08–2.54)**	**2.28 (1.56–3.33)**
12 Months	**0.27 (0.19–0.38)**	1.02 (0.71–1.45)	0.81 (0.54–1.21)	0.99 (0.69–1.41)
Age				
<10 years	**0.34 (0.27–0.43)**	**0.38 (0.31–0.47)**	**0.29 (0.24–0.36)**	**0.32 (0.26–0.39)**
10–14 years	0.92 (0.65–1.30)	0.96 (0.69–1.32)	0.97 (0.71–1.34)	1.05 (0.77–1.44)
15–45 years	Ref	Ref	Ref	Ref
>45 years	1.36 (0.93–1.98)	0.83 (0.60–1.16)	0.79 (0.57–1.09)	0.88 (0.64–1.19)
Gender				
Male	Ref	Ref	Ref	Ref
Female, non-pregnant	0.97 (0.81–1.16)	0.95 (0.0–1.13)	0.89 (0.75–1.06)	**0.73 (0.62–0.87)**
Pregnant female	**0.28 (0.15–0.51)**	**0.48 (0.27–0.85)**	**0.33 (0.19–0.56)**	0.54 (0.29–1.01)

## Discussion

Coverage surveys are an attractive option for validation of MDA coverage because, unlike “reported” coverage (reported by drug distributors), they are not affected by the accuracy of drug distributor records or target population estimates. However, the value of coverage survey estimates is dependent, among other things, on the ability and willingness of those surveyed to accurately report their treatment history. The intent of this study was to assess recall accuracy among persons receiving PCT after an integrated MDA for LF, schistosomiasis, onchocerciasis, and STH in Togo, in a coverage survey conducted shortly after the MDA. A secondary aim was to assess the duration of recall accuracy up to a year after the MDA.

We found that survey respondents in Binah District, Togo, reported with high accuracy whether they had taken MDA medications when interviewed at one month post-MDA, and that the survey-derived coverage estimates for taking at least one of the MDA medications were remarkably consistent at 1, 6, and 12 months. Unfortunately, difficulty finding persons in the treatment registers make it difficult to draw conclusions about the accuracy of recall at 6 and 12 months. As expected, concordance for the question of whether a person had received MDA medications (“Did you take at least one of the pills the CHW offered you?”) was highest one month after MDA and lowest at 12 months, but was >86% for all surveys. This high concordance was not simply a function chance agreement based on independently high treatment rates and survey-reported coverage. For example, expected random concordance for the question “Did you take at least one of the pills?”, given the observed survey response rates (87–91%) and the MDA register records (80–88% treatment), would be 79%, 81%, and 74% for the 1-, 6-, and 12-month surveys, yet the actual concordance rates observed were significantly higher (95%, 92%, and 86%, respectively; kappa statistic p<0.001 for all comparisons).

By both self-report and MDA register, over 80% of the studied population took at least one MDA medicine, and this proportion among the eligible population was even higher. Furthermore, nearly everyone who took at least one pill took all the medications offered them. This suggests that a single-question query about MDA participation could accurately predict (in Togo) whether each individual medication was taken. In other settings where there is a high degree of resistance to taking MDA medications [11], misrepresentation due to social desirability bias (i.e. reporting that one took medications when in fact one did not) may result in lower concordance. One indication that mothers in our surveys generally sought to be truthful rather than automatically reporting that their children were treated (due to social desirability bias), is that mothers did not over-report treatment of children <2 years of age (S3 Table).

Our study has important strengths and limitations. It is the first study to directly measure the accuracy of respondent recall in the setting of MDA for NTDs. Because the study was conducted after an integrated MDA that distributed three medications, it provided an opportunity to probe respondent recall not only for overall MDA participation, but also for recall of specific medications. In addition, surveying three independent samples of the same population, but at different times, provided the opportunity to study consistency of survey responses with increasing time from MDA. To our knowledge, this is the first study of coverage survey recall to specifically address this issue. Several limitations have been discussed, including the possibility that the 6-month survey data may overestimate concordance for individual pill recall. In addition, it is likely that the MDA register data presented here under-represent the true MDA coverage in the population in the 6- and 12-month surveys. The field teams reported difficulties finding treatment data in the MDA registers (which were kept by the CHW, not the study teams) for many persons surveyed in the later surveys. Because persons not found in the MDA register were assumed to be untreated, the MDA register results likely underestimate treatment and therefore overestimate discordance in these surveys. In this respect, it is likely that the survey responses for overall MDA participation (taking at least one pill or taking all pills) are more accurate that the MDA register responses. All three surveys estimated the value of the same parameter—coverage in Kémérida Canton in the last MDA. As would be expected in accurate estimates of the value of this parameter, the three recall-based estimates were very close to each other. The drop in coverage based on the treatment registers is therefore likely due to the increasing failure rate, over time, to find the names of persons in the registers who were in the survey sample and said they had been treated. This hypothesis is further supported by the distribution of discordant responses, which were distributed relatively equally between those falsely reporting treatment and those falsely reporting no treatment at the 1-month survey, but were heavily skewed towards those reporting treatment but not recorded as being treated in the later surveys (Table 4).

It is important to point out that coverage surveys (and population-based surveys in general) are useful only to the extent that the survey population is representative of the general population of interest. Our systematic sampling of a high proportion (one eighth to one fourth) of the population of compounds in Kémérida Canton should have created very representative surveys of the Canton. Whether Kémérida Canton is representative of all areas under MDA in Togo, or of other areas of world, is important to consider when evaluating our results. In addition, a small proportion (5–12%, Table 1) of compounds selected could not be interviewed, and an additional 12–15% of persons in interviewed compounds were excluded from analysis either because they were not present at the MDA or because they were not present during the survey. Because persons not interviewed might be more likely to have missed MDA (due to frequent absence from the compound), it is possible that the MDA coverage estimates from our survey responses slightly overestimate true coverage. However, there is no reason to believe those not surveyed would have been less likely to accurately report whether they received MDA medications.

Despite its limitations, this study provides several important insights. First, coverage survey responses were highly stable between 1, 6, and 12 months, suggesting that, when necessary, coverage surveys in this population might be delayed up to a year to allow inclusion in larger, periodic, multipurpose surveys such as the Demographic and Health Survey [12] without adversely affecting coverage estimates. Second, in a setting where MDA compliance is high, a single question, such as “Did you take all the medications offered you during the MDA” may be sufficient to accurately estimate coverage for multiple medications. Third, it seems clear that respondents recall more accurately for themselves than for their children—at least in the setting where both parent and child are being treated. Fourth, pregnancy can be a particularly difficult confounder when it comes to both appropriate receipt of MDA medication and to accurate reporting in coverage surveys. Announcing a pregnancy is a cultural taboo in Togo [13] and our data suggest pregnant women may choose to be treated rather than disclose they are pregnant by refusing MDA [14]. Similarly, women who are treated when pregnant may be less likely to accurately report treatment.

Finally, our results highlight some of the difficulties of conducting field studies in resource-poor settings, and suggest several potential improvements for future studies of recall accuracy, including (1) blinding of surveyors to MDA treatment register results to prevent inappropriate correcting or correlation of responses by the study team; (2) documentation of treatment status of all household members at the time of MDA, including those not treated; and (3) the use of a unique identifier that can be used to facilitate unambiguous matching of survey respondents to their corresponding record in the MDA register. Subsequent studies have incorporated these improvements [15]. Further studies in a setting where MDA compliance is historically lower, will be very helpful in validating the use of cluster-sample surveys for verification of post-MDA PCT coverage.

## Supporting Information

S1 Table Demographics of those living in Kémérida during the 2008 MDA, but who were unavailable for interview. (DOCX)

S2 Table Concordance for pill number recall. (XLSX)

S3 Table Survey responses for several MDA quality indicators. (XLSX)

S1 Checklist STROBE. (PDF)
