# Supplementary material for: Accuracy of Coverage Survey Recall following an Integrated Mass Drug Administration for Lymphatic Filariasis, Schistosomiasis, and Soil-Transmitted Helminthiasis
Source: PLoS Negl Trop Dis. 2016 Jan 14;10(1):e0004358. doi: 10.1371/journal.pntd.0004358 (PMC4713198; doi:10.1371/journal.pntd.0004358)
Supplement: S1 Table — (DOCX) [file pntd.0004358.s001.docx]

**S1 Table.**

| **Supplemental Table 1: Demographics of those in surveyed compounds excluded from analysis, by reason for exclusion** | | | | |
| --- | --- | --- | --- | --- |
|  | | **Survey** | | |
|  |  | **1 Month** | **6 Months** | **12 Months** |
| A. Persons present in Kémérida during the 2008 MDA, but who were unavailable for interview (for children aged <10 years, no adult responsible for the child was available) | | | | |
|  | Records excluded | 73 | 187 | 17 |
|  | Gender |  |  |  |
|  | Male | 29 (40%) | 83 (44%) | 9 (53%) |
|  | Female | 20 (27%) | 95 (51%) | 7 (41%) |
|  | No gender reported | 24 (33%) | 9 (5%) | 1 (6%) |
|  | Median age in years (IQR) | 21 (12-32) | 19 (12-35) | 22 (8-29) |
|  | Age <10 years | 6 (10%) | 32 (17%) | 4 (24%) |
|  | Age <1 year | 0 (0%) | 0 (0%) | 0 (0%) |
| B. Persons not present in Kémérida during the 2008 MDA* | | | | |
|  | Records excluded | 19 | 16 | 94 |
|  | Gender |  |  |  |
|  | Male | 10 (53%) | 6 (37%) | 36 (38%) |
|  | Female | 9 (47%) | 10 (63%) | 58 (62%) |
|  | No gender reported | - |  | - |
|  | Median age in years (IQR) | 22 (13-30) | 18 (3-27) | 7 (0-22) |
|  | Age <10 years | 3 (16%) | 6 (37%) | 49 (52%) |
|  | Age <1 year | - | 1 (6%) | 28 (30%) |
| C. Persons neither present in Kémérida during the 2008 MDA, nor available for interview (for children aged <10 years, no adult responsible for the child was available) | | | | |
|  | Records excluded | - | 2 | 15 |
|  | Gender |  |  |  |
|  | Male | - | 2 (100%) | 5 (33%) |
|  | Female | - | - | 10 (67%) |
|  | No gender reported | - | - | - |
|  | Median age in years (IQR) |  | 19 | 17 (5-28) |
|  | Age <10 years | - | - | 6 (40%) |
|  | Age <1 year | - | - | 2 (13%) |
| *Although the accuracy of the responses cannot be verified, it is interesting to note that 87% of those reportedly not present at MDA reported taking no MDA medications (100%, 94%, and 83% at 1, 6, and 12 months, respectively) | | | | |
